# Mitigating sarcoplasmic reticulum stress limits disuse-induced muscle loss in hindlimb unloaded mice

**DOI:** 10.1038/s41526-022-00211-w

**Published:** 2022-07-11

**Authors:** Amir Ali Khan, Muhammad Tehsil Gul, Asima Karim, Anu Ranade, Muhammad Azeem, Zeinab Ibrahim, Gopika Ramachandran, Vidhya A. Nair, Firdos Ahmad, Adel Elmoselhi, Rizwan Qaisar

**Affiliations:** 1 Department of Applied Biology, College of Sciences, University of Sharjah, Sharjah, 27272 UAE; 2 Human Genetics & Stem Cells Research Group, Research Institute of Sciences & Engineering, University of Sharjah, Sharjah, 27272 UAE; 3 Department of Basic Medical Sciences, College of Medicine, University of Sharjah, Sharjah, 27272 UAE; 4 Department of Applied Physics and Astronomy, College of Sciences, University of Sharjah, Sharjah, 27272 UAE; 5 Cardiovascular research group, Sharjah Institute for Medical Research, University of Sharjah, Sharjah, 27272 UAE

**Keywords:** Physiology, Disease genetics

## Abstract

Muscle disuse in the hindlimb unloaded (HU) mice causes significant atrophy and weakness. However, the cellular and molecular mechanisms driving disuse-muscle atrophy remain elusive. We investigated the potential contribution of proteins dysregulation by sarcoplasmic reticulum (SR), a condition called SR stress, to muscle loss during HU. Male, c57BL/6j mice were assigned to ground-based controls or HU groups treated with vehicle or 4-phenylbutyrate (4-PBA), a potent inhibitor of SR stress, once a day for three weeks. We report that the 4-PBA reduced the SR stress and partly reversed the muscle atrophy and weakness in the HU mice. Transcriptome analysis revealed that several genes were switched on (*n* = 3688) or differentially expressed (*n* = 1184) due to HU. GO, and KEGG term analysis revealed alterations in pathways associated with the assembly of cilia and microtubules, extracellular matrix proteins regulation, calcium homeostasis, and immune modulation during HU. The muscle restoration with 4-PBA partly reversed these changes along with differential and unique expression of several genes. The analysis of genes among the two comparisons (HU-v *vs*. control and HU-t *vs*. HU-v.) shows 841 genes were overlapped between the two comparisons and they may be regulated by 4-PBA. Altogether, our findings suggest that the pharmacological suppression of SR stress may be an effective strategy to prevent disuse-induced muscle weakness and atrophy.

## Introduction

Mechanical unloading of skeletal muscle results in rapid loss of muscle mass and force with time^1^. This condition is relevant to a plethora of scenarios, from prolonged bed rest in patients with chronic pathologies^2,3^ to microgravity during space flights^4^. Several interventions (dietary supplements, electrical stimulation, passive muscle contraction, and physical exercise) have been used to reduce disuse muscle atrophy^5^. However, technical challenges and poor compliance with these therapies warrant the necessity of a pharmacological intervention to boost muscle mass and force during prolonged inactivity.

The experimental rodent model of hindlimb unloading (HU) recapitulates several features of mechanical unloading, including muscle atrophy and weakness^6^, and is an excellent model to test potential pharmacological interventions^7^. However, no drug therapy exists to effectively prevent muscle atrophy and weakness, partly because the exact molecular mechanism(s) coupling inactivity to muscle impediment are poorly understood. The emerging role(s) of endo/sarcoplasmic reticulum (ER/SR) in skeletal muscle diseases is only beginning to surface^8,9^. The SR plays a pivotal role in calcium homeostasis and protein folding in the mammalian skeletal muscle. However, overload of misfolded and/or unfolded proteins in the SR lumen, a condition called SR stress, can lead to pathological consequences in chronic diseases^10^. SR stress leads to the activation of a signaling network called the unfolded protein response (UPR) and its downstream consequences. Chronic elevation of UPR activates cell death pathways (apoptosis, inflammation, and autophagy) associated with degenerative muscle disorders, including aging, myopathies, and catabolic conditions^11^. However, establishing a direct causality between SR stress and muscle detriment requires assessing muscle mass and force by mitigating SR stress in disease models. To our knowledge, only a few studies have investigated the effects of inhibition of SR stress on skeletal muscle in disease conditions, none of them involve disuse atrophy. Thus, the treatment of mice with 4-phenyl butyrate (4-PBA), a pan-SR stress inhibitor, ameliorates muscle atrophy in burn injury^12^ or genetic mutations associated with SR calcium dysregulation^13^. However, a recent study shows that short-term inhibition of SR stress exacerbates rather than protects against cancer cachexia^14^. These discrepancies warrant a thorough investigation of molecular alterations in mechanically unloaded skeletal muscle with 4-PBA treatment. However, such a characterization has not been performed before. Therefore, a careful dissection of global transcriptomic changes in skeletal muscle is required to characterize the contribution of SR stress to skeletal muscle impairment.

Here, we investigated the potential contribution of SR stress to muscle weakness and atrophy in HU mice. We show that pharmacological inhibition of SR stress in HU conditions partially restores muscle mass and strength. Partial muscle restoration is associated with alteration in signature transcriptomic changes in skeletal muscle. Using a non-biased approach, we identified several candidate genes and molecular pathways that dictate SR stress-induced muscle restoration in HU conditions. Our findings indicate that pharmacological inhibition of SR stress may be a promising therapy for disuse-induced muscle loss.

## Methods

### Hindlimb unloading (HU) mice model

**Fig. 1:**
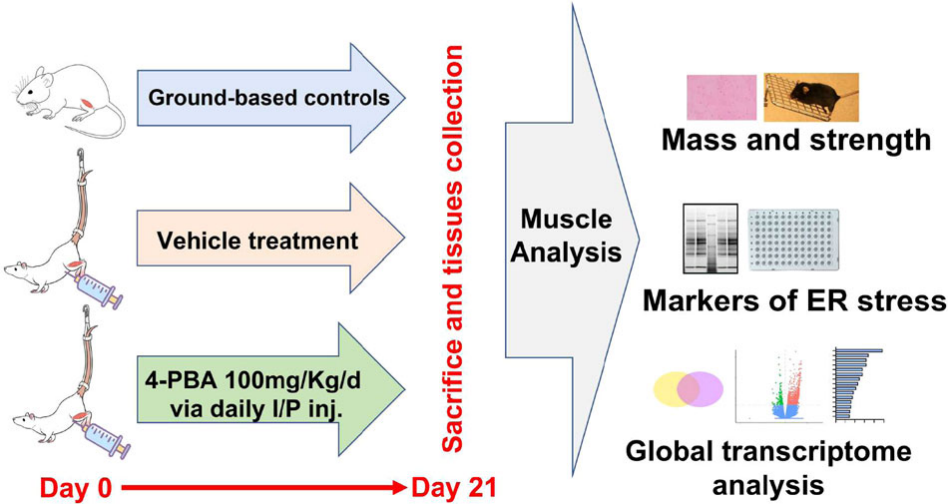
Experimental design. Experimental design of the study.

We randomly assigned 4-month-old, male c57BL/6j mice into ground-based controls (*n* = 10) and HU mice. HU mice were further divided into two groups and treated with either PBS as the vehicle (HU-v; *n* = 10) or 4-PBA (HU-t; 100 mg/kg/d for three weeks via intraperitoneal injections) (*n* = 8). The dose was chosen based on published literature^15^. Mice were kept under controlled environmental conditions (20 ± 1 °C, with light/dark periods of 12 h each) with food (standard chow diet for mice) and water provided ad-libitum. As previously described, the HU mice were suspended for 21 days in specially designed cages^16,17^. At the end of the experiments, mice were euthanized via cervical dislocation, and the gastrocnemius muscles were immediately excised, weighed, and snap-frozen in liquid nitrogen for further analysis, as described previously^18^ (Fig. 1). The experimental protocol was approved by the University of Sharjah ACUC (Animal Care and Use Committee) in agreement with accepted international standards. All methods were carried out in accordance with the relevant guidelines and regulations. Additionally, all methods are reported in accordance with ARRIVE guidelines.

### Grip strength measurements

We measured the grip strength using a Grip Strength Meter with a mesh grid pull bar (Columbus Instruments, Columbus, OH) specifically designed for mice. Mice were allowed to grip the metal grids of the grip meter with their paws and gently pulled backward in a horizontal plane until they could no longer hold the grip. The grip strength was obtained from the two forelimbs and all four limbs, including hindlimbs. The peak grip strength obtained in ten consecutive trials was designated as the mouse’s grip strength and was normalized to body weights, as described previously^17^.

### Sample collection and Library preparation

Total RNA was extracted from gastrocnemius muscles of the three groups of mice. Before fragmentation, the mRNAs were purified with magnetic beads tagged with Ploy-T tail. The first strand was prepared with the random hexamers primers, and then the second strand was also synthesized. The library was checked with Qubit and quantified with real-time PCR. Next, the library was analyzed with a bioanalyzer for size detection. The libraries were pooled, and then they were sequenced on Illumina platforms.

### Clustering and sequencing

The samples were index-coded, and clusters were generated, followed by sequencing the library using an Illumina platform to generate paired-end reads.

### Quality control

Raw reads in the FASTQ format were processed by fastp. To get the clean data, reads containing the adapter sequence and low quality were removed. Following the clean reads, Q20 and Q30, clean data were calculated. Further downstream analysis was calculated using clean data with good quality. Using the Spliced Transcripts software (STAR), paired-end clean reads were aligned to the reference genome. This alignment is based on the RNAseq alignment algorithm followed by seed clustering and stitching. Supplementary file 1 contains the QC statistics of the data, while Supplementary File 2 contains the mapping statistics.

### Quantification

The numbers of reads that were mapped to each gene were counted using the FeatureCounts. To normalize the reads, Fragments Per Kilobase of transcript sequence per Million (FPKM) fragments mapped were calculated^19^.

### Differential expression analysis

Differential genes expression analysis was performed between two conditions/groups (three biological replicates per condition) using DESeq2 R package. The *P* values were calculated using Benjamini and Hochberg’s method for the false discovery rate (FDR). Genes with an adjusted *P*-value < 0.05 were assigned as significantly differentially expressed genes.

### Gene ontology (GO), KEGG pathways, and reactome enrichment analysis

Biological ontologies is a common way for finding shared functions among genes. GO enrichment analysis of differentially expressed genes was performed by the clusterProfiler R package. GO terms with corrected *P*-value less than 0.05 (Fisher exact test) were considered significantly enriched in the differential expressed genes. For the KEGG enrichment, R package clusterProfiler was used to find KEGG pathways that were statistically significant among the differential expression genes, as described previously^20^. KEGG terms with adjusted *P* value less than 0.05 (Fisher exact test) were considered significant enrichment. For reactome enrichment analysis, the clusterProfiler was also used and terms with adjusted *p*-value less than 0.05 were considered significantly enriched.

### Quantitative real-time PCR validation

A set of 13 randomly selected genes were chosen for RT-PCR to validate the transcript data. The procedure is described in detail elsewhere^21^. Briefly, total RNA was extracted using the RNase kit (Qiagen, Valencia, CA, USA) from 20 mg of frozen gastrocnemius tissues. cDNA was synthesized using QuantiTect Reverse Transcriptase kit (Qiagen 205,311, Germany), and quantitative real-time PCR was performed using SYBR Green PCR Master Mix with primers. The primer sequence is described in Table 1. Calculations were performed using delta-delta-ct method, as described previously^22^.

**Table 1:** Primers’ sequence. Sequence of the primers used in the study.

Primer name	Forward	Reverse	Reference
s-XBP1	CTGAGTCCGAATCAGGTGCAG	GTCCATGGGAAGATGTTCTGG	^44^
u-XBP1	CAGCACTCAGACTATGTGCA	GTCCATGGGAAGATGTTCTGG	^44^
ATF4	GGGTTCTGTCTTCCACTCCA	AAGCAGCAGAGTCAGGCTTTC	^45^
CHOP	CCACCACACCTGAAAGCAGAA	AGGTGAAAGGCAGGGACTCA	^45^
ADAM1B	TTCCCTCCATGAGGAATACG	GTGCCTTCCTCTTTGCAGTC	^46^
PEG3	GGTTCAGTGTGGGTGCACTAGACT	GCTCACACCCAAGGGCTTGAGCGT	^47^
H2Q7	CGGGCCAACACTCGCTGCAA	GTATCTGCGGAGCGACTGCAT	^48^
NLE1	TATCAAGCTGTGGGATGGC	AGCATATACCTCATCGGCGT	^49^
MEG3	GGACTTCACGCACAACACGTT	GTCCCACGCAGGATTCCA	^50^
ELN	GGAGTTCCCGGTGGAGTCTATT	ACCAGGAATGCCACCAACACCTG	^51^
SCARF1	CTCTCCAGAGGTGCTCAACC	ATGCCTCCATCAGTGGTCTC	^52^
UBXN10	GAGTCTGTGCAACGGTCT CA	TCCTGGCTTGAATCCTCTTG	Self-designed primer
HSF5	GCTGTAGGACAATTTCACCGG	TTCCAAGGGAGTTCTGCCAC	^53^
EID3	AGGAGGAGGAAGGCTCAGAC	GCCTCTCTGGTTCTGCTCAC	^54^
NME8	GACGATGCGGTTAAGGTCTC	TTGCCTCTGCATCAGTATGG	Self-designed primer
ZDHHC19	TTGCTGCCTTCAATGTAACG	TGAGAAGTTGAGCGAGACGA	Self-designed primer
HSPA1B	CAAGATCACCATCACCAACG	ATGACCTCCTGGCACTTGTC	^55^
GAPDH	CATCACTGCCACCCAGAAGACTG	ATGCCAGTGAGCTTCCCGTTCAG	^56^

(s-XBP1; spliced x-box-binding protein1, u-XBP1; unspliced x-box-binding protein1, ATF4; activating transcription factor4; CHOP; C/EBP homologous protein, ADAM1B; ADAM Metallopeptidase Domain 1B, PEG 3; paternally expressed 3, H2Q7; histocompatibility 2, Q region locus 7, NLE1; notchless protein homolog 1, MEG3; maternally expressed 3, ELN; elastin, SCARF1; Scavenger Receptor Class F Member 1, UBXN10; UBX domain protein 19, HSF5; heat shock transcription factor 5, EID3; EP300 Interacting Inhibitor Of Differentiation 3, NME8; NME family member 8, ZDHHC19; Zinc Finger DHHC-Type Palmitoyltransferase 19, HSPA1B; heat shock protein family A member 1B).

### Western blot

Muscle tissues were homogenized in RIPA buffer containing 50 mM Tris (pH = 7.4), 150 mM NaCl and protease inhibitors. Proteins were quantified using the Bio-Rad kit (Sigma-Aldrich, Poole, UK) and transferred to a nitrocellulose membrane after electrophoresis using 8–15% polyacrylamide gels, as described before^23^. The western blot was derived from the same experiment and the tissues were processed in parallel. The western blot was derived from the same experiment and the tissues were processed in parallel. Membranes were probed using anti-GRP78 primary antibody (Cat # 3177, Cell Signaling Technology) at 1:1000 dilution and a secondary antibody at 1:10,000 (HRP-linked anti-rabbit IgG; Cat # 7074 S, cell signaling, Danvers, MA 01923, USA) as described previously^22^.

### Histology

Gastrocnemius muscles were immediately embedded in optimal cutting temperature (OCT) compound and snap frozen. Sections were cut at 10 µm with a Leica 3050 cryotome and stained for hematoxylin and Eosin, as previously described^18^. Zeiss LSM 510 Meta confocal microscope (Melville, NY, USA) was used for imaging and the Image J software (National Institute of Health, Bethesda, MD, USA) was used for image analysis, as described previously^18^.

### Raman spectroscopy

The experimental Raman spectra were obtained by using Renishaw inVia confocal Raman microscope. Three specimens of gastrocnemius muscles were selected from each of the three groups of mice. We collected ten spectra from randomly selected locations of each sample to obtain the average. Each recording involved exposure of a 50 µm muscle segment for 10 s to a 785 nm laser of 1% intensity (14 mW). The spectral range was kept between 100 and 1200 cm^−1^, representing the peaks for biological molecules. All spectra were collected at the intervals of 500 cm^−1^ and then stitched together to obtain the full spectrum.

### Statistical analysis

All numerical values are presented as mean ± SEM, and the comparisons among the groups were performed by one-way analysis of variance (ANOVA) and Turkey’s multiple comparison tests, with a single pooled variance. For the real time PCR, unpaired student’s t-test was performed. Data were analyzed using GraphPad Prism 9 (GraphPad Software, La Jolla, CA), and *p* < 0.05 was considered statistically significant.

### Reporting summary

Further information on research design is available in the Nature Research Reporting Summary linked to this article.

## Results

### 4-PBA partially restored muscle mass and strength in HU conditions

**Fig. 2:**
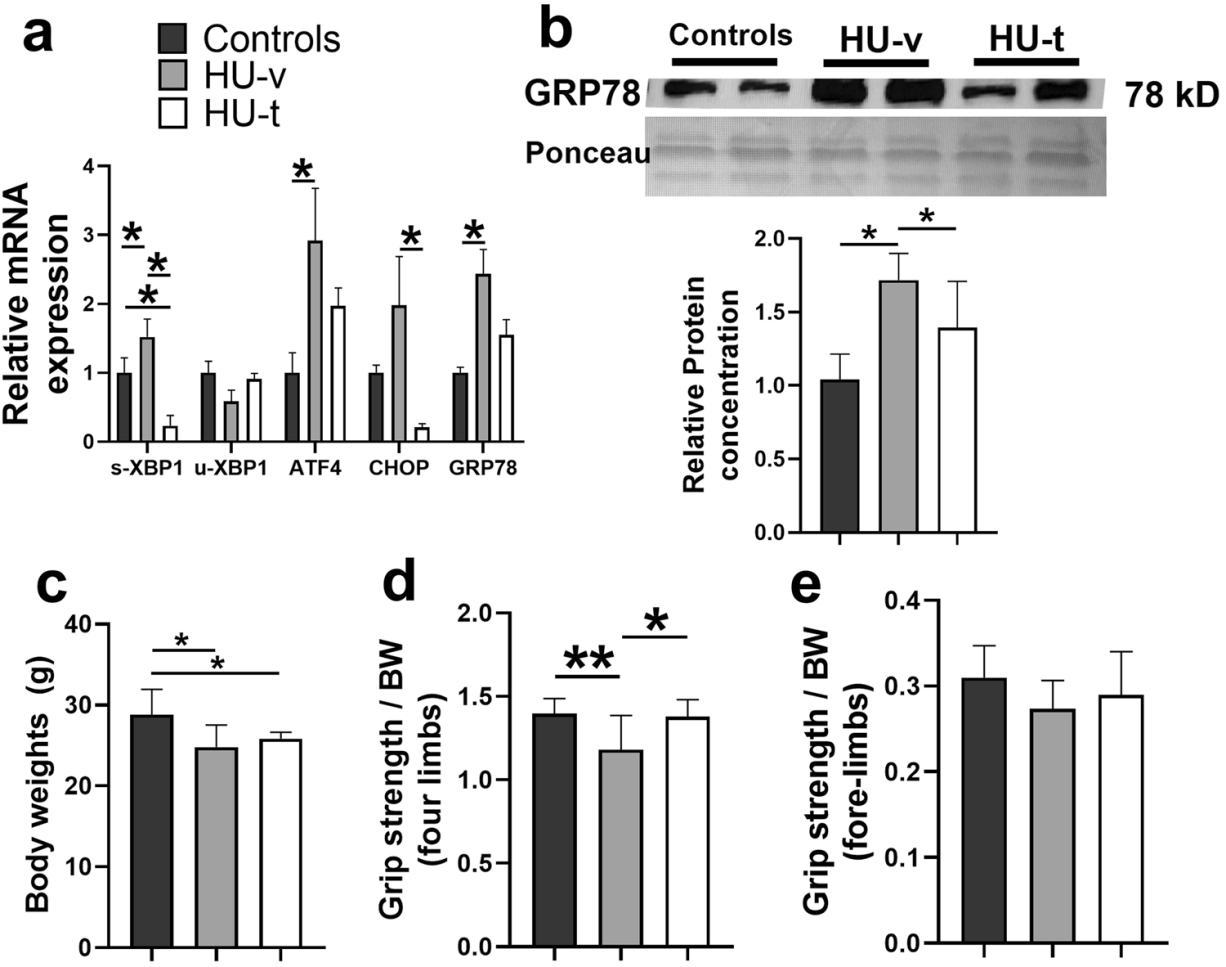
Mice characterization. Relative mRNA (**a**) and protein (**b**) expression of markers of SR stress (A), body weights (**c**), relative grip strength of all four limbs (**d**) and frontal forelimbs (**e**) in ground-based control and the hindlimb-unloaded mice treated with vehicle (HU-v) or 4-PBA (HU-t). Data is presented as mean ± SEM; One way analysis of variance, *p < 0.05 (*n* = 8–11/group). (s-XBP1; spliced x-box-binding protein1, u-XBP1; unspliced x-box-binding protein1, ATF4; activating transcription factor4; CHOP; C/EBP homologous protein, GRP78; glucose regulatory protein 78).

**Fig. 3:**
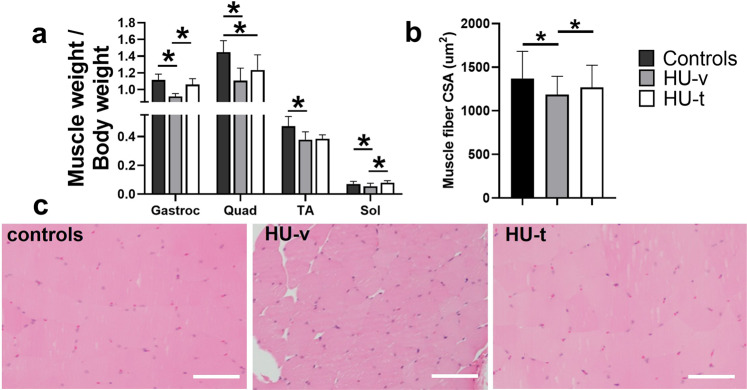
Muscle mass and fibers size. The relative masses of hindlimb muscles, including gastrocnemius, quadriceps, tibialis anterior (TA), and soleus (**a**), and single fiber cross-sectional areas (**b**) and hematoxylin & eosin images (**c**) of the gastrocnemius muscles in ground-based control and the hindlimb-unloaded mice treated with vehicle (HU-v) or 4-PBA (HU-t). Data is presented as mean ± SEM; One way analysis of variance, *p < 0.05 (*n* = 6–11/group) (scale bar = 50 µm).

We first confirmed the activation of SR stress following HU and its subsequent inhibition with 4-PBA treatment (Fig. 2a, b). Accordingly, the signature markers of SR stress exhibited an HU-induced-upregulation, which was prevented with 4-PBA treatment. The HU mice showed significantly reduced body weights irrespective of their treatment status than the ground-based control mice (*p* < 0.05, one-way ANOVA) (Fig. 2c). The HU also reduced the grip strength of all four limbs and frontal forelimbs when normalized to body weight (*p* < 0.05, one-way ANOVA) (Fig. 2d, e). 4-PBA treatment restored the grip strength of four limbs to normal levels (*p* < 0.05, one-way ANOVA) (Fig. 2d). The HU also reduced the relative muscle masses of several hindlimb muscles, including gastrocnemius, quadriceps, tibialis anterior, and soleus (Fig. 3a). Treatment with 4-PBA restored the relative muscle weights of gastrocnemius, quadriceps, and soleus muscles to normal levels (all *p* < 0.05, one-way ANOVA) (Fig. 3a). Similarly, the mean single fiber cross-sectional area of the gastrocnemius was reduced with HU and restored with the treatment by 4-PBA (Fig. 3b, c).

### The uniquely and differentially expressed genes following HU and treatment with 4-PBA

We next investigated the muscle transcriptome analysis from the three groups. First, a quality check of the transcript data was performed. Supplementary file 1 contains the quality of the sequence data, while Supplementary File 2 includes the summary of the mapping data. These data indicates that several sequencing reads were uniquely mapped on the genome; the total mapping rate was more than 90% for every sample. We also validated the randomly selected genes through RT-PCR to validate the transcriptome data (Supplementary Fig. 1a). We also found an upregulation of several antioxidant genes in the HU-t group, indicating the potential of 4-PBA to counter oxidative stress (Supplementary Fig. 1b).

We report a unique expression of 3688 and 429 genes in HU-v and control groups, respectively (Supplementary Fig. 2a). 4-PBA treatment resulted in a unique expression of 1906 genes compared to controls (Supplementary Fig. 3b) and 1228 genes compared to HU-v mice (Supplementary Fig. 3c). We also enumerate several uniquely expressed genes in different comparisons (Supplementary Fig. 2a–c). These genes are primarily related to calcium regulation, mitochondrial function, SR organization, and cilia assembly, indicating the critical role(s) of these processes during muscle impediment and recovery. All the uniquely expressed genes among the three comparisons are included in Supplementary File 3.

**Fig. 4:**
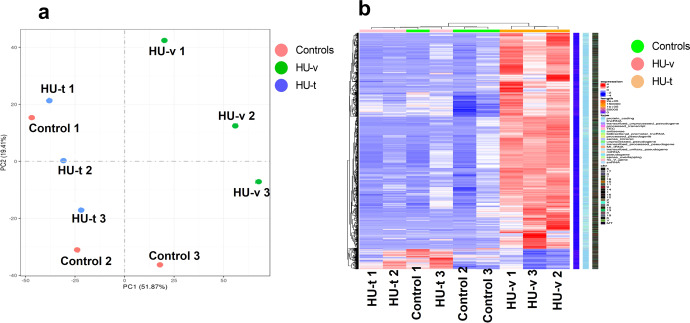
PCA and the heat map. The principal component analysis (PCA) analysis (**a**) and the heat map (**b**) of genes expressed in ground-based control and the hindlimb-unloaded mice treated with vehicle (HU-v) or 4-PBA (HU-t). The upper genes in the heat map are upregulated (red) in the HU-v group. However, the lower genes are downregulated in the HU-v group and are upregulated in the HU-v and HU-t groups (*n* = 3/group).

**Fig. 5:**
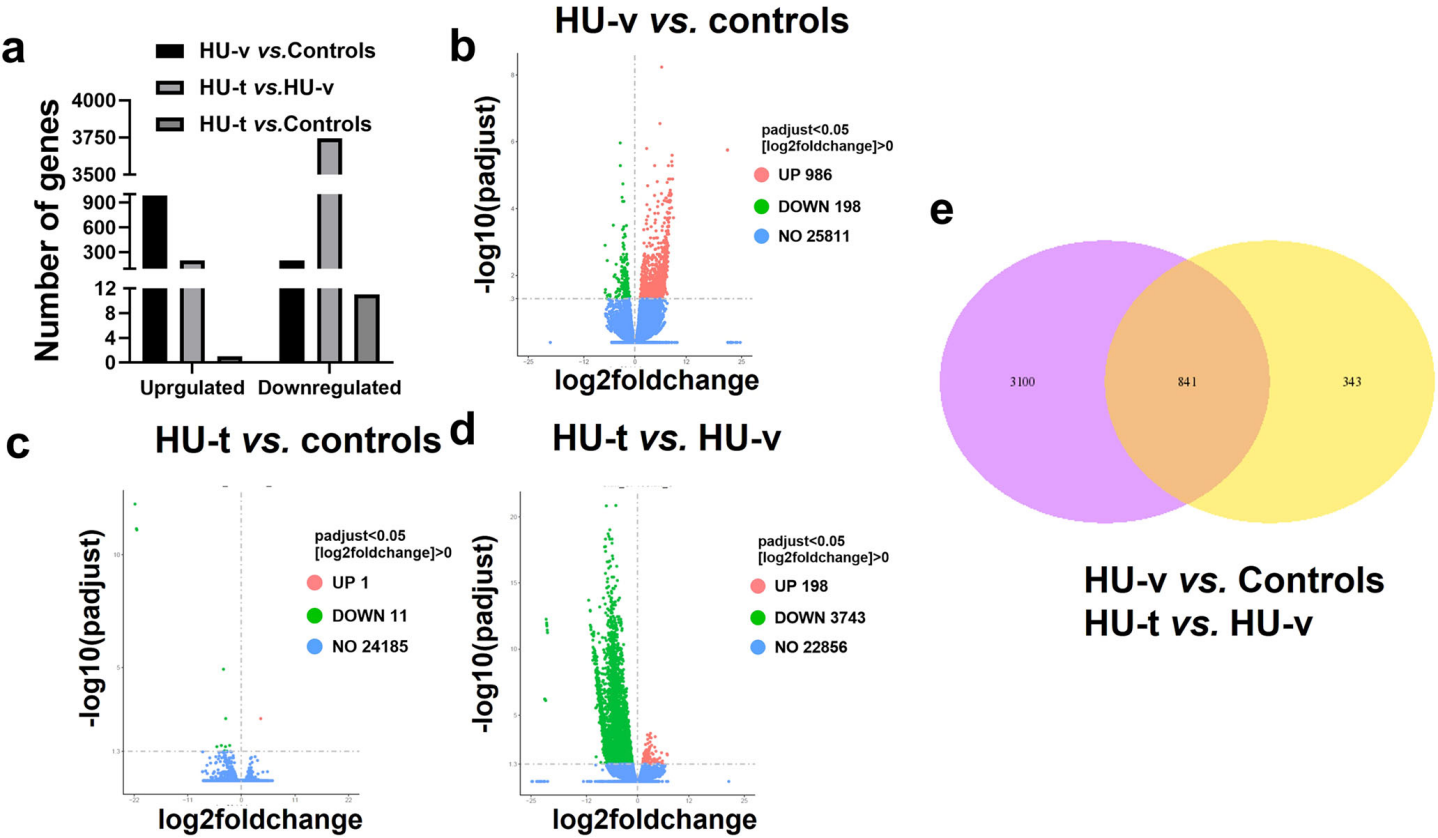
Differentially expressed genes. Summary (**a**), the volcano plots (**b**–**d**), and Venn diagrams (**e**) of the differential expressed genes in ground-based control and the hindlimb-unloaded mice treated with vehicle (HU-v) or 4-PBA (HU-t) (A). The horizontal axis in the Volcano plots represent the fold change of genes in different samples, while the vertical axis represents statistically significant degrees of changes in gene expression levels; the smaller the corrected p-value, the bigger -log10 (corrected p-value), the more significant the difference. The points represent genes, and blue dots indicate no significant difference in genes, red dots indicate upregulated differential expression genes, green dots indicate downregulated differential expression genes (*n* = 3/group).

**Fig. 6:**
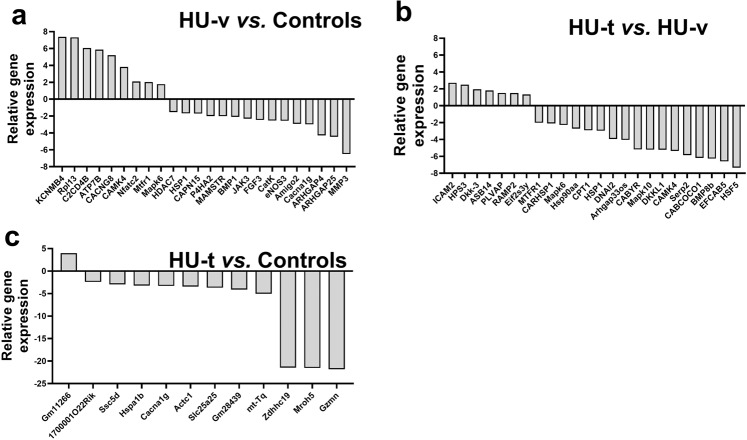
Differentially expressed genes. Transcriptome data revealing the alterations in differentially expressed genes in paired comparisons between HU-V *vs*. control (**a**), HU-t *vs*. HU-v (**b**), and HU-t *vs*. control (**c**) groups (*n* = 3/group). (HU-v; hindlimb-unloaded mice treated with vehicle, HU-t; hindlimb-unloaded mice treated with 4-PBA, KCNMB4; potassium large conductance calcium-activated channel subfamily M, beta member 4, opposite strand 2, Rpl13; ribosomal protein L13 pseudogene, C2CD4B; C2 calcium-dependent domain containing 4B, ATP7B ATPase; Cu++ transporting beta polypeptide, CACNG8; calcium channel voltage-dependent gamma subunit 8, CAMK4; calcium/calmodulin-dependent protein kinase IV, Nfatc2; nuclear factor of activated T cells, cytoplasmic calcineurin dependent 2 interacting protein, Mtfr1; mitochondrial fission regulator 1, Mapk6; mitogen-activated protein kinase 6, HDAC7; histone deacetylase 7, HSP1; heat shock protein 1, CAPN15; calpain 15, P4HA2; procollagen-proline 2-oxoglutarate 4-dioxygenase alpha II polypeptide, MAMSTR; MEF2 activating motif and SAP domain containing transcriptional regulator, BMP1; bone morphogenetic protein 1, JAK3; Janus kinase 3, FGF3; fibroblast growth factor receptor 3, CatK; cathepsin K, eNOS3; nitric oxide synthase 3 endothelial cell, Amigo2; adhesion molecule with Ig like domain 2, Cacna1g; calcium channel, voltage-dependent T type alpha 1G subunit, ARHGAP4; Rho GTPase activating protein 4, ARHGAP25; Rho GTPase activating protein 25, MMP3; matrix metallopeptidase 3, ICAM2; intercellular adhesion molecule 2, HPS3; HPS3 biogenesis of lysosomal organelles complex 2 subunit 1, Dkk-3; dickkopf WNT signaling pathway inhibitor 3, ASB14; ankyrin repeat and SOCS box-containing 14, PLVAP; plasmalemma vesicle associated protein, RAMP2; receptor (calcitonin) activity modifying protein 2, Eif2s3y; eukaryotic translation initiation factor 2 subunit 3 structural gene Y-linked, MTFR1; mitochondrial fission regulator 1, CARHSP1; calcium regulated heat stable protein 1, Mapk6; mitogen-activated protein kinase 6, Hsp90aa; heat shock protein 90, alpha (cytosolic) class A member 1, CPT1; carnitine palmitoyltransferase 1c, HSP1; heat shock protein 1-like, DNAI2; dynein, axonemal, intermediate chain 2, Arhgap33os; Rho GTPase activating protein 33 opposite strand, CABYR; calcium-binding tyrosine-(Y)-phosphorylation regulated (fibrousheathin 2), Mapk10; mitogen-activated protein kinase 10, DKKL1; dickkopf-like 1, CAMK4;calcium/calmodulin-dependent protein kinase IV, Serp2; stress-associated endoplasmic reticulum protein family member 2, CABCOCO1; ciliary associated calcium binding coiled-coil 1, BMP8b; bone morphogenetic protein 8b, EFCAB5; EF-hand calcium binding domain 5, HSF5; heat shock transcription factor family member 5, Gm11266; predicted gene 11266, 1700001O22Rik; RIKEN cDNA 1700001O22 gene, Ssc5d; scavenger receptor cysteine rich family 5 domains, Hspa1b; heat shock protein 1B, Cacna1g; calcium channel voltage-dependent T type alpha 1G subunit, Actc1;actin alpha cardiac muscle 1, Slc25a25, solute carrier family 25 (mitochondrial carrier, phosphate carrier) member 25, Gm28439; predicted gene 28439, mt-Tq; mitochondrial encoded tRNA glutamine, Zdhhc19; zinc finger DHHC domain containing 19, Mroh5; maestro heat-like repeat family member 5, Gzmn; granzyme N).

Before we conducted the differential expression analysis, PCA and hierarchical clustering analysis were performed on the gene expression in the three conditions (Fig. 4a, b). The analysis revealed that the genes expression due to HU are different and a significant number of genes are expressed following HU. The expression of genes following the 4-PBA treatment reversed the transcriptomes as most of genes expressed in the control and treated samples are clustered in PCA and exhibited similar expressions in the hierarchal clustering. The differential expression analysis revealed that 986 genes were upregulated while 198 were downregulated in the skeletal muscle of HU-v *vs*. control mice (Fig. 5a, b). However, treatment with 4-PBA reduced the number of differentially expressed genes (Fig. 5c). We report 3,941 differentially regulated genes in addition to 1646 uniquely expressed genes between HU-v and HU-t muscles, indicating the vast transcriptomic changes induced by 4-PBA in partial muscle restoration (Fig. 5a, d). These findings also show the molecular interface of SR stress with muscle atrophy pathways in conditions of HU. All of the differential expressed genes are listed in the Supplementary File 4. The Venn diagram shows the overlap of genes among the two comparisons (HU-v *vs*. control and HU-t *vs*. HU-v) (Fig. 5e). We found an overlap of 841 genes between the two comparisons. These findings indicate the potential expression of genes to reverse the muscle atrophy under HU conditions with 4-PBA treatment. Several candidate genes were differentially regulated in paired comparisons across groups (Fig. 6a–c). These genes are primarily associated with cellular calcium regulation, mitochondrial maintenance and biogenesis, cell stress pathways, and the structural integrity of muscle fibers.

### GO and KEGG term analysis for identification of significantly altered pathways following HU and treatment with 4-PBA

**Fig. 7:**
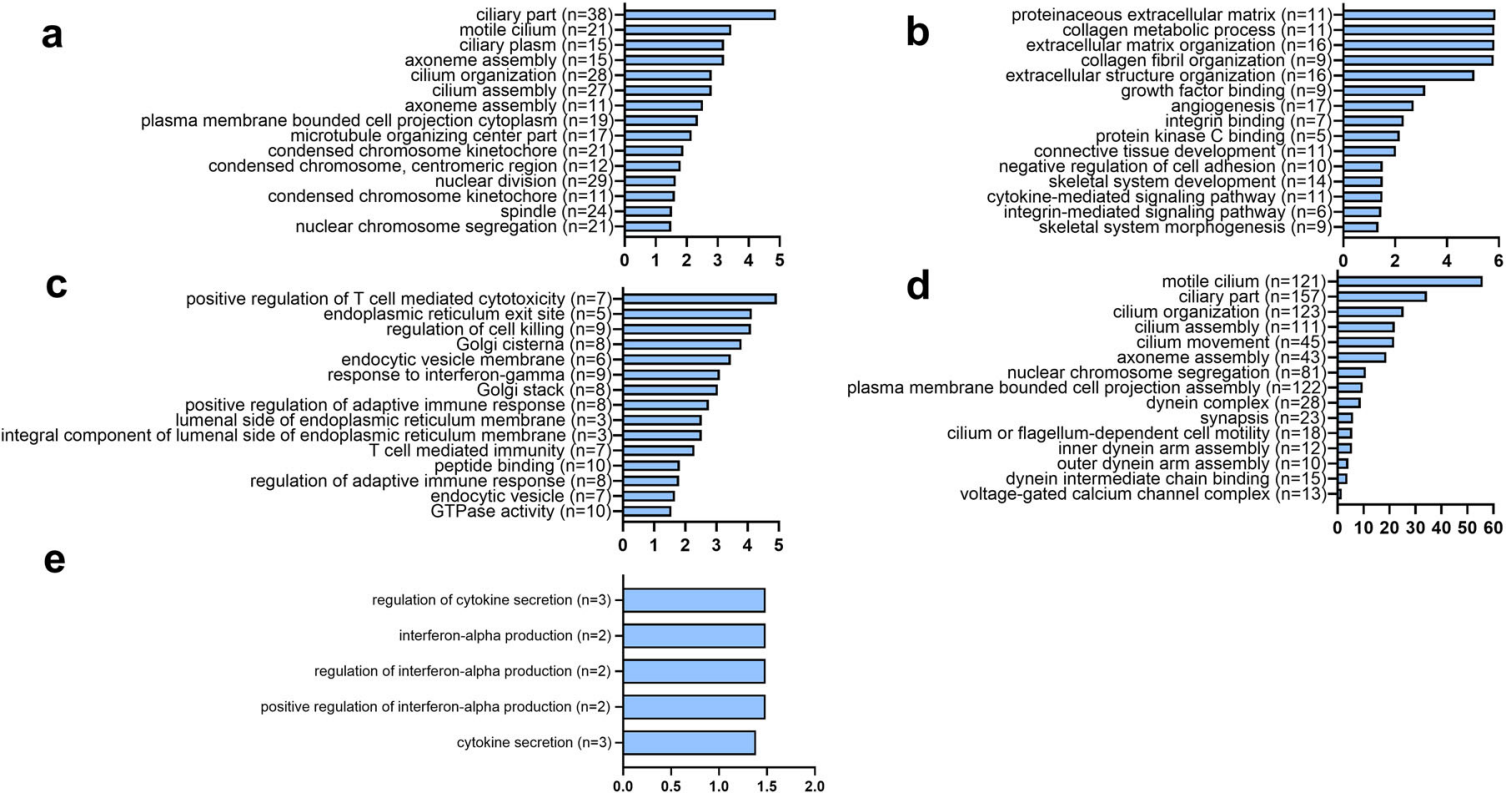
Cellular pathways based on GO terms enrichment analysis. GO term analysis describing significantly up- (**a**) and downregulated (**b**) GO terms in the hindlimb-unloaded mice treated with vehicle (HU-v), when compared to ground-based controls group, up- (**c**) and downregulated (**d**) terms in the hindlimb-unloaded mice treated with 4-PBA (HU-t), when compared to HU-v group, and upregulated (**e**) terms in HU-t mice than ground-based control groups. There was no significantly downregulated pathway in the HU-t group than ground-based control mice. The horizontal axis indicates the −log of adjusted *p* values, while the numbers in parenthesis indicate the number of significantly altered genes for individual pathways (*n* = 3/group).

GO enrichment analysis was carried out on differentially expressed genes (Fig. 7a–e). The primary and relevant GO terms enriched in the downregulated genes in HU-v muscles compared to control primarily related to extracellular matrix (ECM) remodeling (Fig. 7b). ECM-related KEGG pathway was also enriched in the downregulated genes compared with control. Additionally, other pathways involving PI3-AKT, JAK-STAT, and AGE-RAGE signaling were also enriched in the downregulated genes in HU-v muscles compared with control (Fig. 7b). The differentially upregulated genes associated with GO terms analysis are mainly cell cycle-related (Fig. 7a).

**Fig. 8:**
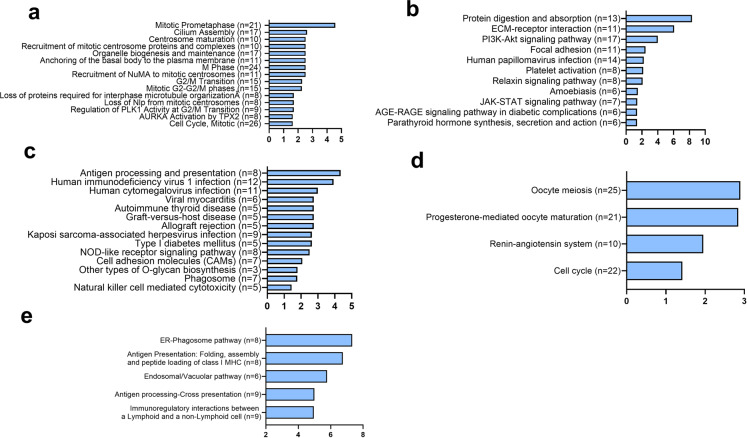
Cellular pathways based on KEGG and reactome enrichment analysis. Main and related KEGG terms and reactome enrichment analysis describing significantly up- (**a**) reactomes and downregulated (**b**) KEGG pathways in the hindlimb-unloaded mice treated with vehicle (HU-v), when compared to ground-based controls group, up- (**c**) and downregulated (**d**) KEGG pathways in the hindlimb-unloaded mice treated with 4-PBA (HU-t), when compared to HU-v group. 7E: The critical reactome enrichment in upregulated genes in HU-t compared HU-v (**e**). There was no significant KEGG pathway nor any reactome enriched in the HU-t with ground-based control. The horizontal axis indicates the −log of adjusted *p* values, while the numbers in parenthesis indicate the number of significantly altered genes for individual pathways (*n* = 3/group).

We also carried out KEGG terms and reactome enrichment analysis on differentially expressed genes (Fig. 8a–e). The most significant and relevant results are reported. There was no KEGG pathways enrichment in the differentially regulated genes in the HU-t muscles compared with control, indicating the positive effects of 4-PBA on HU muscles. Only upregulated genes in 4-PBA treated muscles have been associated with a few functions, such as cytokine secretion indicating the regulation of exocytosis by the 4-PBA. The main GO terms that were enriched in upregulated genes in HU-t muscles than HU-v muscle were mainly immunity-related terms (Fig. 7c), while the GO terms enriched in the downregulated genes in this comparison were sperm-related. It seems the 4-PBA treatment has drastic effects on the transcriptomic profiling with alterations in apparently irrelevant genes. The reactomes that were enriched between control and vehicle were mainly associated with cell cycle regulation (Fig. 8a). The KEGG pathway enrichment further confirmed the immunity-related pathways that were enriched in upregulated genes in HU-t muscles compared with HU-v muscle (Fig. 8c). The reactome analysis indicated the ER-phagosome pathway was upregulated in 4-PBA treated HU muscles compared with vehicle-treated HU muscle (Fig. 8e). All the significant GO terms, KEGG, and reactomes are included as Supplementary Files 5 and 6.

### 4-PBA prevented Raman spectroscopic changes in HU conditions

The Raman spectroscopic analysis was carried out to investigate the global molecular phenotypes of the muscle tissues under various conditions. The comparison of the control, HU-v, and HU-t muscles revealed major differences in the locations and magnitudes of multiple peaks, indicating qualitative and quantitative changes in the molecular compositions of skeletal muscles (Supplementary Fig. 3). We found a shift in the peak at 159 cm^−1^ for the control muscles to 151 cm^−1^ in the HU-v muscle. Similarly, the intensity of the peak at 220 cm^−1^ was higher in the HU-v sample. The intensities of peaks at the 344 and 412 cm^−1^ were significantly reduced in HU-v muscles compared to the control, along with the appearance of a new peak at 460 cm^−1^, indicating conformational changes in the asparagine and the glutamine molecules. Treatment with 4-PBA reversed some of the changes in Raman spectra associated with HU. Additionally, based on published literature^24–26^, the bands at 159, 223, 344, and 1209 cm^−1^ relate to asparagine^24^, while asparagine has an overlap with glutamine at the frequencies 412, 533, and 551 cm^−1^. We also found that the hydroxyproline, UDP- D- glucose, and tryptophan together contribute to the peaks at 710 and 755 cm^−1^^25^. Among other amino acids, tryptophan was represented at 1004 cm^−1^^26^, tyrosine at 595 cm^−1^ and the proline at 936, 1046, 1090, 1125, 1166, and 1245 cm^−1^. Additionally, the twisting and bending of the carbon–hydrogen bonds, CH_3_ and CH_2_ of praline and proline molecules contribute to the bands at 1343, and 1450 cm^−1^^26^. Likewise, the shoulders at 1557 and 1645 cm^−1^ were due to carbon–carbon bond stretching in the praline molecule^26^.

## Discussion

We report a pathological role of SR stress in disuse-induced muscle loss. Briefly, muscle atrophy and weakness following three weeks of HU are associated with a pathological increase in SR stress. Conversely, 4-PBA inhibited the SR stress and partially restored the muscle mass and strength during HU. Transcriptomic analysis unraveled several candidate pathways and processes that may have a potential role in SR stress-induced muscle loss.

A critical function of the elevated SR stress is to induce cell death pathways, contributing to muscle atrophy and weakness^27^. Disuse atrophy of skeletal muscle is a common consequence of mechanical unloading but is poorly understood at molecular and cellular levels. Additionally, the contribution of SR stress to disuse-induced muscle atrophy is not well known. An earlier study found no alterations in the expression of SR stress markers following one week of HU^28^. However, two weeks of HU was associated with an upregulation of SR chaperons BIP and PDI in the rat skeletal muscle^29^.

Interestingly, these effects were more pronounced in the fast-twitch than slow-twitch muscles. Due to ultrastructural differences, the fast-twitch fibers are more vulnerable to damage and atrophy than slow-twitch fibers^30^. We carefully selected predominantly fast-twitch gastrocnemius muscle and three weeks duration of HU to obtain significant muscle pathology for characterization of SR stress and related signaling pathways.

Our transcriptomic data revealed several candidate pathways as the potential mediators of muscle loss due to elevated SR stress. Of particular interest are the pathways or terms associated with the assembly, organization, and motility of cilia. The emerging roles of cilia in maintaining and regulating muscle satellite cells are increasingly being recognized^31^. For example, ablation or pharmacological inhibition of cilia inhibits satellite cell proliferation and differentiation in in vitro^32^ and mice^33^. The GO terms and reactome analysis revealed that unloading causes regulation of the cell cycle and proliferation. The GO term cilium assembly was enriched in the upregulated gene in the HU-v muscle. The other primary GO term enriched in the upregulated genes in HU-v are related to the microtubules indicating the cytoskeletal proteins are affected in the muscle during the HU conditions. In the downregulated genes, the main GO terms enriched are associated with the ECM. Hence, it indicates that the HU affects the expression of cytoskeletal as well as ECM protein. The main KEGG pathways enriched in the downregulated genes are PI3k-Akt, focal adhesion, and Jak-Stat pathways. Further study is warranted to find the regulation of these pathways and their effects on the ECM and atrophy in the muscles. One study suggested that the microgravity induces muscle atrophy by downregulating genes that are involved in muscle differentiation and development. However, the roles of differential and uniquely expressed genes during mechanical unloading warrant further investigations.

Our transcriptomic data also revealed alterations of several pathways associated with cilia structure, assembly, organization, motility, and maintenance with 4-PBA treatment, which may partly explain the restoration of muscle mass and strength in these mice. We found a reduction of SR stress and its downstream UPR pathways with 4-PBA treatment, which is consistent with the restoration of cilia^34^. Thus, the 4-PBA may potentially mitigate muscle loss by restoring the cilia population in the satellite cells. The comparison between the differential expressed gene between the two comparisons - HU-v *vs*. control and HU-t *vs*. HU-v- shows 841 genes are overlapped between the two comparisons indicating these genes may be regulated by 4-PBA. These genes are involved in the reversing the muscle atrophy.

The HU-induced upregulation of pathways associated with muscle fibrosis was not observed in the mice treated with 4-PBA. Previous studies have reported increased muscle fibrosis in conditions of mechanical unloading^35^. Several candidate mechanisms are associated with muscle fibrosis in multiple pathologies^36,37^. Additionally, the role of myofibroblasts in muscle fibrosis is well characterized^38^. The cilia of myofibroblasts contain several elements of the TGF-β pathway, which contribute to signaling associated with fibrosis. The satellite cells can also modulate muscle fibrosis by altering the extracellular microenvironment and secreting matrix proteins^31^. Thus, it seems alterations in cilia signaling may contribute to muscle fibrosis in unloading conditions. The association of ER stress with fibrosis is established in multiple tissues^39^ but is poorly characterized in skeletal muscle. However, several lines of evidence point towards an association between SR stress and muscle fibrosis. For example, SR stress is increased in myopathies due to defects in collagen VI synthesis^40^. Similarly, SR stress is elevated in muscular dystrophies, with fibrosis as a hallmark feature^9^.

The SR in skeletal muscle also specializes in calcium homeostasis. The coupling between protein folding and calcium regulatory functions of SR has been suggested so that elevated SR stress leads to SR calcium dysregulation^41^. We found HU-induced alterations in several genes related to calcium homeostasis, which were partially reversed with 4-PBA treatment. These findings confirm and extend the earlier reports of the functional interface between SR protein and calcium regulation.

The reduction in peak intensities of asparagine and glutamate molecules correspond to their reduced concentration. Interestingly, the reduced quantity of both molecules is associated with muscle atrophy. Asparagine suppresses the expression of atrophy markers^42^, while glutamate has anti-catabolic properties^43^. 4-PBA induced partial restoration of these molecules, which may contribute to the attenuation of atrophy in HU-t mice. In addition, we also found conformational changes in the structures of these amino acids, which may have functional implications by reducing their activities.

We did not investigate the off-target effects of 4-PBA on other body organs. An elevated SR/ER stress is implicated in pathologies of multiple body organs, including bone tissues, during HU^27^. Owing to the well-established crosstalk of skeletal muscle with bone and other body organs, it is possible that the 4-PBA may partly preserve skeletal muscle through systemic effects.

Taken together, we have shown that pharmacological mitigation of SR stress may be a potent therapy to reduce muscle decline due to mechanical unloading. We also propose the alterations in multiple signaling pathways as candidate mediators of 4-PBA-induced muscle recovery during mechanical unloading. Our findings show that 4-PBA can be an effective therapy to prevent and/or reduce disuse atrophy and weakness of skeletal muscle. Future studies may be required to investigate further and validate the mechanistic link between mitigation of SR stress and muscle recovery during mechanical unloading.

## Supplementary information

Reporting Summary

Supplementary file with figure legends

Supplementary file 1.

Supplementary file 2.

Supplementary file 3.

Supplementary file 4.

Supplementary file 5.

Supplementary file 6.

## Data Availability

All data generated analyzed during this study have been deposited in the GEO database NCBI, with the accession number GSE189455.
