# Supplementary material for: Mitigating sarcoplasmic reticulum stress limits disuse-induced muscle loss in hindlimb unloaded mice
Source: NPJ Microgravity. 2022 Jul 11;8:24. doi: 10.1038/s41526-022-00211-w (PMC9273600; doi:10.1038/s41526-022-00211-w)

Supplementary Information

Mitigating sarcoplasmic reticulum stress limits disuse- induced muscle loss in hindlimb unloaded mice

Amir Ali Khan[1], Muhammad Tehsil Gul[1], Asima Karim[2], Anu Ranade[2], Muhammad Azeem[3], Zainab Ibrahim[4], Gopika Ramachandran[4], Vidhya A Nair[4], Firdos Ahmad[2,4], Adel Elmoselhi[2,4], *Rizwan Qaisar[2,4]

[1]Department of Applied Biology, College of Sciences, University of Sharjah, Sharjah 27272, UAE

[2]Department of Basic Medical Sciences, College of Medicine, University of Sharjah, Sharjah 27272, UAE

[3]Department of Applied Physics and Astronomy, College of Sciences, University of Sharjah 27272, UAE

[4]Cardiovascular research group, Sharjah Institute for Medical Research, University of Sharjah, Sharjah 27272, UAE

Corresponding author
Rizwan Qaisar, MBBS. Ph.D.
Department of Basic Medical Sciences
College of Medicine
University of Sharjah, Sharjah, UAE
Phone: +971 6 505 7254
***ORCID:* 0000-0001-8485-7172**
E-mail: rqaisar@sharjah.ac.ae

Figures legends for supplementary files
Supplementary figure 1.

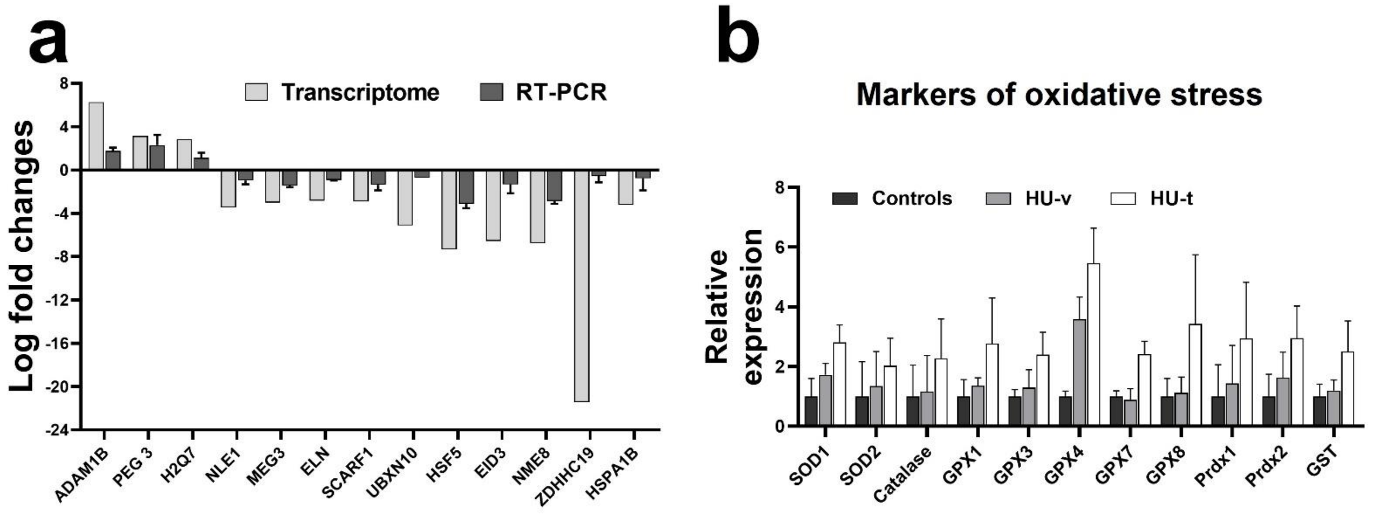

Supplementary figure 1.

Title: Validation of transcriptome data.

Caption: Transcriptome and mRNA expression of randomly selected genes (a) and the transcriptome expressions of the markers of oxidative stress (b) in ground-based controls and the hindlimb-unloaded mice treated with vehicle (HU-v) or 4-PBA (HU-t). Data is presented as mean ± SD, (n = 3 / group).

(ADAM1B; ADAM Metallopeptidase Domain 1B, PEG 3; paternally expressed 3, H2Q7; histocompatibility 2, Q region locus 7, NLE1; notchless protein homolog 1, MEG3; maternally expressed 3, ELN; elastin, SCARF1; Scavenger Receptor Class F Member 1, UBXN10; UBX domain protein 19, HSF5; heat shock transcription factor 5, EID3; EP300 Interacting Inhibitor Of Differentiation 3, NME8; NME family member 8, ZDHHC19; Zinc Finger DHHC-Type Palmitoyltransferase 19, HSPA1B; heat shock protein family A member 1B, SOD1; superoxide dismutase 1, SOD2; superoxide dismutase 2, GPX1; glutathione peroxidase 1, GPX3; glutathione peroxidase 3, GPX4; glutathione peroxidase 4, GPX7, glutathione peroxidase 7, GPX8; glutathione peroxidase 8, Prdx1; peroxiredoxin 1, Prdx2; peroxiredoxin 2, GST; glutathione s-transferase).

Supplementary figure 2.

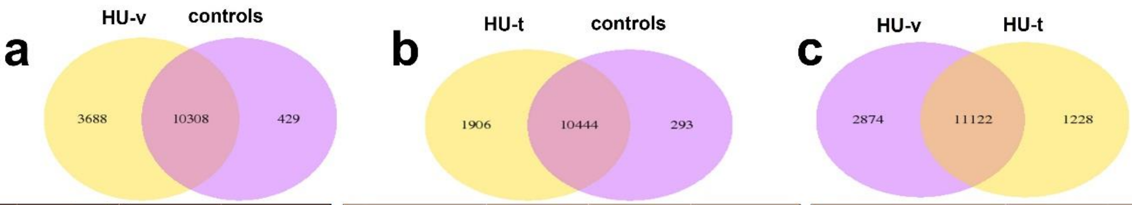

Control mice		
	ID	Name
vs. HU-v	ENSMUSG00000026029	Casp8 (caspase 8)
	ENSMUSG00000026459	Myog (myogenin)
	ENSMUSG00000005102	Eif2ak4 (eukaryotic translation initiation factor 2 alpha kinase 4)
	ENSMUSG00000003352	Cacnb3 (calcium channel, voltage-dependent, beta 3 subunit)
	ENSMUSG00000053137	Mapk11 (mitogen-activated protein kinase 11)
	ENSMUSG00000010830	Kdel3 (KDEL endoplasmic reticulum protein retention receptor 3)
	ENSMUSG00000031710	Ucp1 (uncoupling protein 1)
	ENSMUSG00000048007	Timm8a1 (translocase of inner mitochondrial membrane 8A1)
vs. HU-t	ENSMUSG00000026546	Cfap45 (cilia and flagella associated protein 45)
	ENSMUSG00000035640	Cbarp (calcium channel, beta subunit associated regulatory protein)
	ENSMUSG00000031710	Ucp1 (uncoupling protein 1)
	ENSMUSG00000021556	Golm1 (golgi membrane protein 1)
	ENSMUSG00000090877	Hsp1b (heat shock protein 1B)
	ENSMUSG00000061126	Cyp4f39 (cytochrome P450, family 4, subfamily f, polypeptide 39)
	ENSMUSG00000028310	Ppp3r2 (protein phosphatase 3, regulatory subunit B, alpha isoform)
	ENSMUSG00000026288	Inpp5d (inositol polyphosphate-5-phosphatase D)

Hu-v mice		
	ID	Name
vs. controls	ENSMUSG00000051146	Camk2n2 (calcium/calmodulin-dependent protein kinase II inhibitor 2)
	ENSMUSG00000063704	Mapk15 (mitogen-activated protein kinase 15)
	ENSMUSG00000071229	Timm8a2 (translocase of inner mitochondrial membrane 8A2)
	ENSMUSG00000052584	Serp2 (stress-associated endoplasmic reticulum protein 2)
	ENSMUSG00000034656	Cacna1a (calcium channel, voltage-dependent, alpha 1A subunit)
	ENSMUSG00000019945	Cabcoco1 (ciliary associated calcium binding coiled-coil 1)
	ENSMUSG00000019992	Mtfr2 (mitochondrial fission regulator 2)
	ENSMUSG00000041153	Osgin2 (oxidative stress induced growth inhibitor family member 2)
vs. HU-t	ENSMUSG00000019992	Mtfr2 (mitochondrial fission regulator 2)
	ENSMUSG00000019945	Cabcoco1 (ciliary associated calcium binding coiled-coil 1)
	ENSMUSG00000031837	Necab2 (N-terminal EF-hand calcium binding protein 2)
	ENSMUSG00000052584	Serp2 (stress-associated endoplasmic reticulum protein 2)
	ENSMUSG00000091956	C2cd4b (C2 calcium-dependent domain containing 4B)
	ENSMUSG00000021556	Golm1 (golgi membrane protein 1)
	ENSMUSG00000037386	Rims2 (regulating synaptic membrane exocytosis 2)
	ENSMUSG00000058626	Capn11 (Calpain 11)

Hu-t mice		
	ID	Name
vs. controls	ENSMUSG00000051146	Camk2n2 (calcium/calmodulin-dependent protein kinase II inhibitor 2)
	ENSMUSG00000023050	Map3k12 (mitogen-activated protein kinase kinase kinase 12)
	ENSMUSG00000049281	Scn3b (sodium channel, voltage-gated, type III, beta)
	ENSMUSG00000039543	Cfap70 (cilia and flagella associated protein 70)
	ENSMUSG00000019851	Perp (PERP, TP53 apoptosis effector)
	ENSMUSG00000028578	Caap1 (caspase activity and apoptosis inhibitor 1)
	ENSMUSG00000041153	Osgin2 (oxidative stress induced growth inhibitor family member 2)
	ENSMUSG00000027994	Mcub (mitochondrial calcium uniporter dominant beta subunit)
vs. HU-v	ENSMUSG00000025076	Casp7 (Caspase 7)
	ENSMUSG00000003352	Cacnb3 (calcium channel, voltage-dependent, beta 3 subunit)
	ENSMUSG00000010830	Kdel3 (KDEL endoplasmic reticulum protein retention receptor 3)
	ENSMUSG00000041479	Syt15 (synaptotagmin XV)
	ENSMUSG00000019979	Apaf1 (apoptotic peptidase activating factor 1)
	ENSMUSG00000030669	Calca (calcitonin/calcitonin-related polypeptide, alpha)
	ENSMUSG00000001020	S100a4 (S100 calcium binding protein A4)
	ENSMUSG00000007827	Ankrd26 (ankyrin repeat domain 26)

Supplementary figure 2.

Title: Uniquely expressed genes.

Caption: The number of uniquely expressed genes in the hindlimb-unloaded mice treated with vehicle (HU-v) *vs.* ground-based controls (a), hindlimb-unloaded mice treated with 4-PBA (HU-t) *vs.* ground-based controls (b), and HU-v *vs.* HU-t mice (c). The corresponding tables indicate selected genes that were uniquely expressed in each comparison (n = 8-11 / group). The full list of uniquely expressed genes is available in supplementary table 3.

Supplementary figure 3.

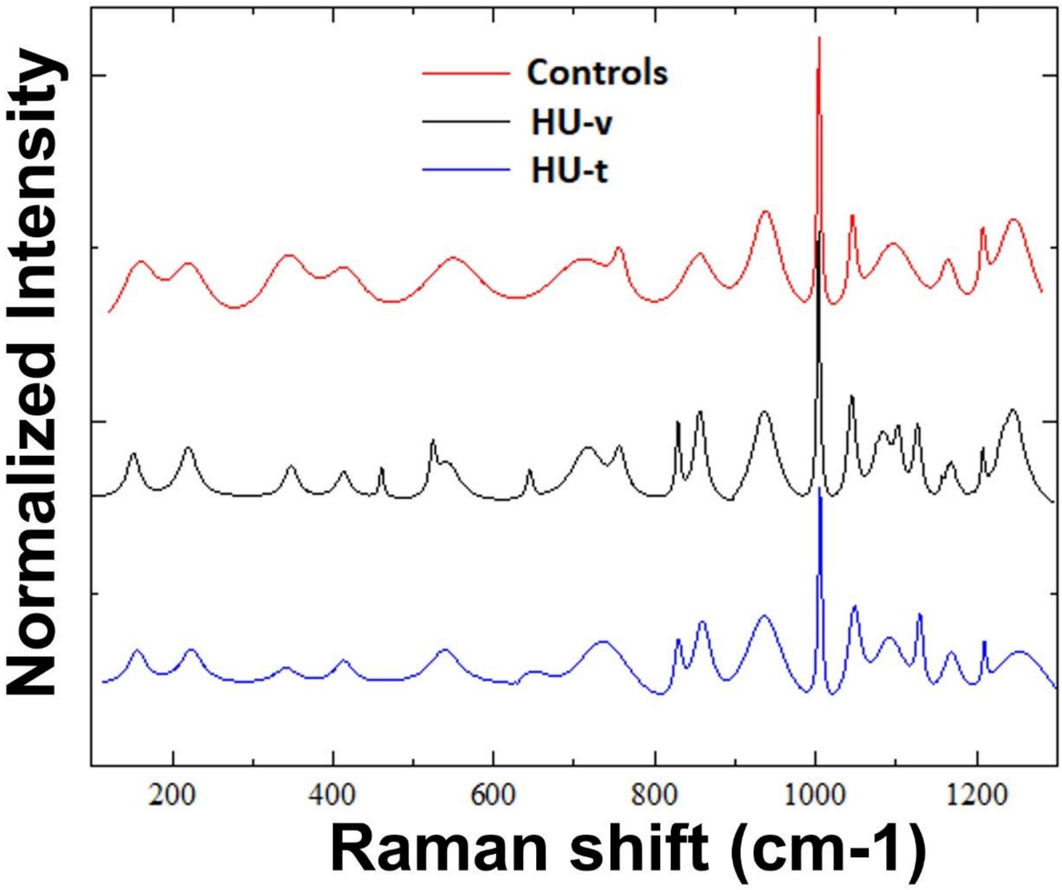

Title: Raman spectroscopy

Caption: Experimentally obtained Raman spectra from the gastrocnemius muscles of the control (red line), HU-v (black line), and HU-t (blue line) mice. (HU-v; hindlimb-unloaded mice treated with vehicle, HU-t; hindlimb-unloaded mice treated with 4-PBA).

Fig 1. Full western blot image.

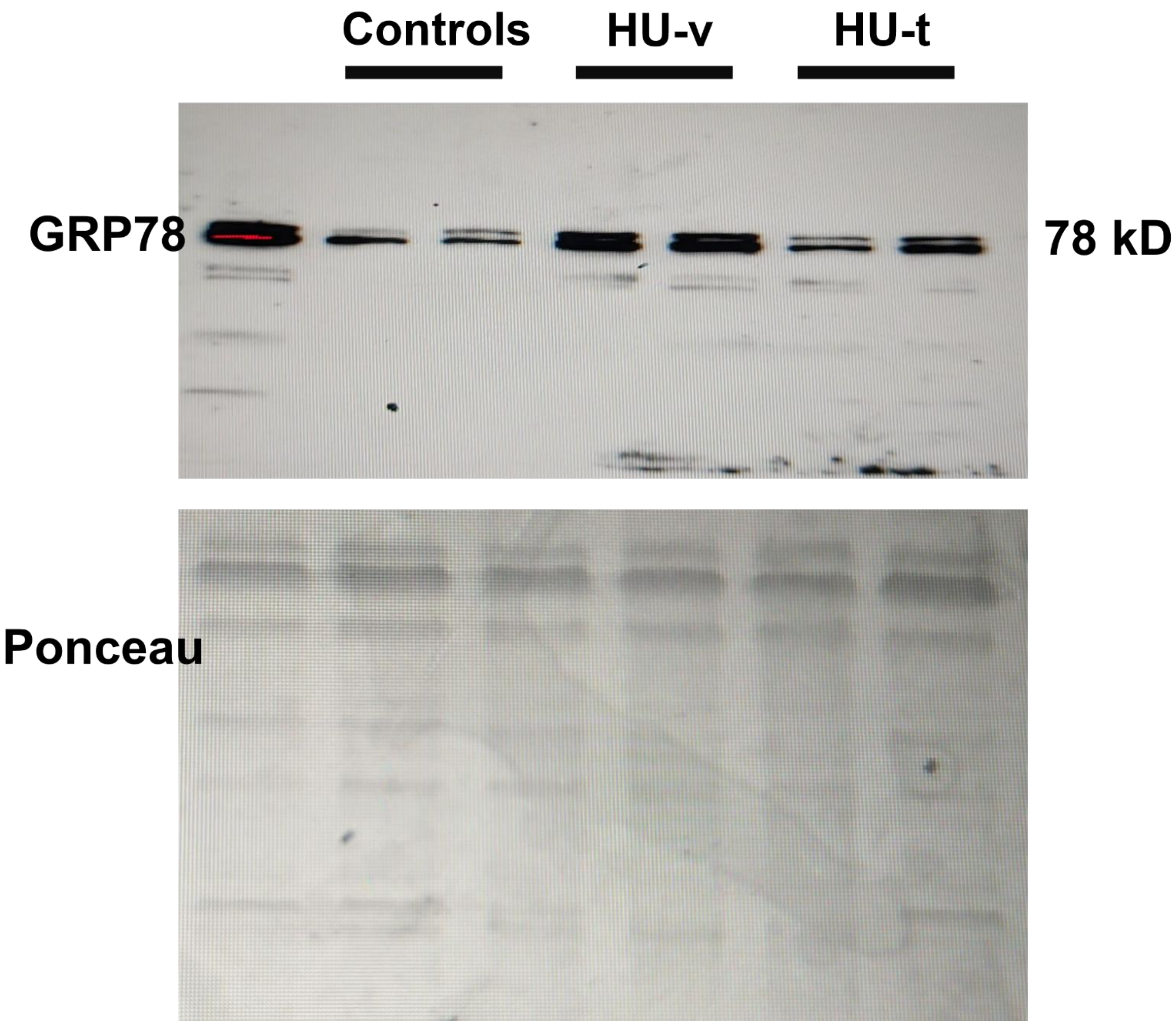

Supplement: Supplementary file 2 — Supplementary file with figure legends [file 41526_2022_211_MOESM2_ESM.pdf]
